# Behavioral evidence for the use of functional categories during group reversal task performance in monkeys

**DOI:** 10.1038/s41598-018-33349-3

**Published:** 2018-10-26

**Authors:** Takayuki Hosokawa, Yasutaka Honda, Munekazu Yamada, Maria del Carmen Romero, Toshio Iijima, Ken-Ichiro Tsutsui

**Affiliations:** 1 Laboratory of Systems Neuroscience, Tohoku University Graduate School of Life Sciences, Sendai, Japan; 2 Department of Sensory Science, Kawasaki University of Medical Welfare, Okayama, Japan; 3 Laboratorium voor Neuro- en Psychofysiologie, KU Leuven Medical School, Leuven, Belgium

## Abstract

A functional category is a set of stimuli that are regarded as equivalent independently of their physical properties and elicit the same behavioral responses. Major psychological theories suggest the ability to form and utilize functional categories as a basis of higher cognition that markedly increases behavioral flexibility. Vaughan claimed the category use in pigeons on the basis of partition, a mathematical criterion for equivalence, however, there have been some criticisms that the evidence he showed was insufficient. In this study, by using a group reversal task, a procedure originally used by Vaughan, we aimed to gather further evidence to prove the category use in animals. Macaque monkeys, which served as subjects in our study, could efficiently perform the task not only with familiar stimulus sets as Vaughan demonstrated but also with novel sets, and furthermore the task performance was stable even when the number of stimuli in a set was increased, which we consider as further evidence for the category use in animals. In addition, by varying the timing of the reversal, we found that a category formation takes place soon after encountering new stimuli, i.e. in a few blocks of trial after a novel stimulus set was introduced.

## Introduction

A functional category is a set of stimuli that are regarded to be equivalent independently of their physical properties and elicit the same behavioral responses, whereas a perceptual category, in contrast, is based on physical similarity among stimuli^1,2^. An important characteristic of functional categories is that once a functional category is formed, if a new contingency (i.e., a response or an outcome) is learned for one member of the category, then that new contingency can be applied to the other members of the category. Thus, the ability to form and use functional categories enables reasoning, which may markedly increase behavioral flexibility. In operational terms, functional categories have been often referred to as equivalence classes or sets. Equivalence class is formally defined as a class capable of satisfying symmetry, transitivity, and reflexivity simultaneously. Sidman has shown that when a verbally competent human subject is trained to match Stimulus A to Stimulus B and B to C, he/she can match B to A and C to B (derived symmetry), A to C (derived transitivity), or A to A, B to B, and C to C (derived reflexivity)^3^. Since then, this ability has been repeatedly confirmed in human subjects^4–6^. On the other hand, many attempts to demonstrate it in animals have been unsuccessful^7,8^.

Vaughan developed another procedure for defining equivalence class on the basis of partition, a mathematical criterion for equivalence^9^. In a task which we address as “group reversal”, pigeons were first trained in concurrent association learning, which was to peck on 20 stimuli out of 40 that were associated with reward and not to peck on the remaining 20 stimuli. After acquisition, the contingencies were reversed for all stimuli, so that previously rewarded stimuli became unrewarded and the previously unrewarded stimuli became rewarded after the reversal. After a large number of reversals, pigeons became capable of adapting to new contingencies after reversal with a few errors. It was explained that the development of rapid adaptations to concurrent association reversals indicates that the pigeons had formed an equivalence class for each of the 20 stimuli. Using this procedure, subsequent studies have shown the formation and use of functional categories in not only pigeons^10^ but also other species, such as dolphins^11^, sea lions^12^, and chimpanzees^13^. However, there have been criticisms for Vaughan’s procedure that it may not necessarily indicate the formation and use of functional categories as in Sidman’s procedure^14,15^. The main point of the criticisms is that, since a quick adaptation at a reversal emerged only after many reversals, animals may learn individual stimulus-response associations and reverse their response based on the individual associations at a reversal. If this is the case, the animals would be able to adapt their response only in a familiar stimulus set, with which they were trained in many repeated reversals, but not in a novel stimulus set, with which they never experienced the reversal. However, it has never been examined on this point using Vaughan’s procedure.

**Figure 1:**
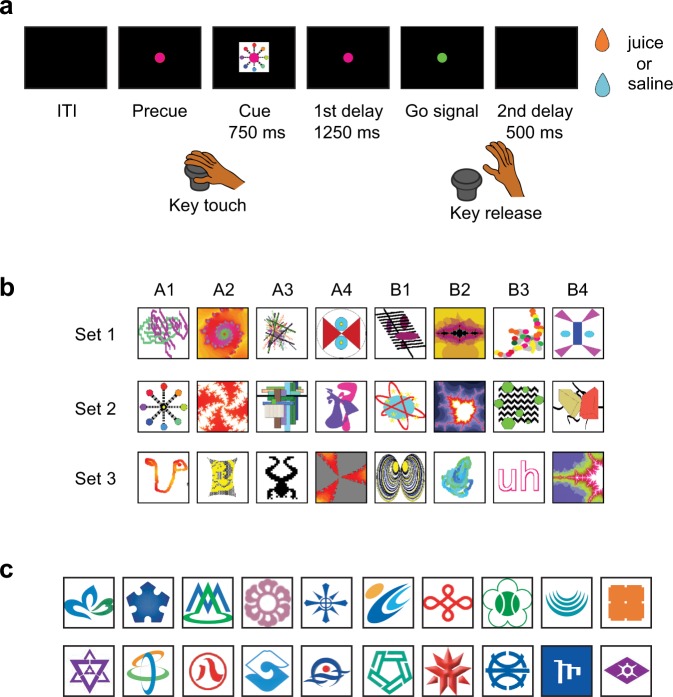
Group reversal task. (**a**) Sequence of events in a trial of the group reversal task. In each trial, a visual stimulus predicted whether the appetitive (fruit juice) or aversive (saline) type of liquid was going to be delivered at the end of the trial. (**b**) Regular stimulus sets used in the group reversal task. Three stimulus sets were used repeatedly during the training. Each set consisted of eight abstract figures. These stimuli were kindly provided by Professor Wolfram Schultz. (**c**) Examples of novel stimuli used in the experiments. Only a portion of novel stimuli used in the experiment are shown here. In each session, 8 novel stimuli (or up to 20 stimuli in Experiment 3) were introduced and the monkeys learned the stimulus–outcome associations.

The purpose of this study was to provide further empirical evidence that would account for the formation and use of a functional category with Vaughan’s procedure, using Japanese monkeys (*Macaca fuscata*) as subjects. We also intended to further specify the characteristics of the functional category established by Vaughan’s procedure. The present study consists of four behavioral experiments. In Experiment 1, to examine whether monkeys would be able to show a quick adaptation at a reversal in Vaughan’s procedure, we trained the monkeys in a group reversal task in which they had to adapt their behavior to a repeated stimulus–outcome reversal with a set of eight distinct visual stimuli and two distinct outcomes (Fig. 1, see Methods). The monkeys were trained with fixed stimulus sets (regular stimulus sets) for several months. In Experiment 2, to examine whether the monkeys learned to utilize a category in the group reversal task, we studied how they would perform the task with a novel stimulus set that was introduced in a daily session. We predicted that if the monkeys had learned a strategy to form a category of stimuli associated with the same outcome in the course of the training with the regular stimulus sets, they would be able to show a quick adaptation even in the first reversal of the sessions with a new stimulus set. In Experiment 3, to further confirm whether the monkeys learned a category to perform the task, we examined how the performance after the reversal would be affected as the number of stimuli in a stimulus set increased. An important characteristic of functional categories is that once a functional category is formed, the memory load to use the category would not be affected by the number of members in the category^16^. Therefore, we predicted that, if they learned to recognize the stimuli predicting the same outcome as a category, the monkeys’ performance at the reversal would not be affected depending on the number of stimuli. In Experiment 4, to study when the category formation is completed after the introduction of a new stimulus set, we examined how the performance after a reversal would be affected as we changed the timing of the reversal with reference to the introduction of a new stimulus set.

## Results

### Experiment 1

**Figure 2:**
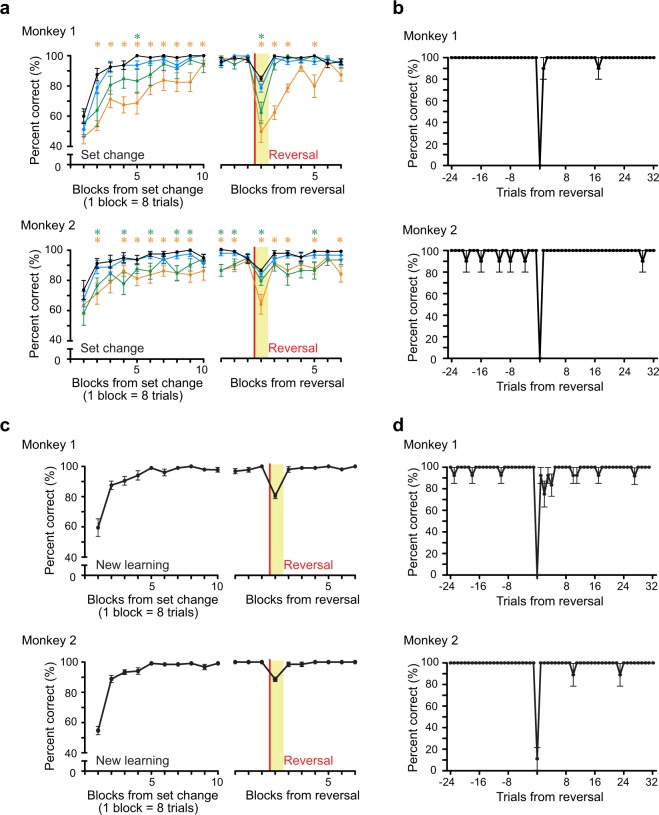
Behavioral performance with regular and novel stimulus sets. (**a**) Blockwise behavioral performance for a regular stimulus set (1 block = 8 trials, mean ± SEM). The left panel shows the performance after a stimulus set change. The right panel shows the performances before and after the reversal. The black line shows the performance in the experimental sessions, while the cyan, green, orange lines show the performance in different divisions of the training sessions after the regular stimulus sets were introduced (cyan: 201st to 300th sessions, green: 101st to 200th sessions, and orange: 1st to 100th sessions). Ten sessions were randomly selected from each division. Green and orange asterisks indicate the performance in green and orange is significantly worse than that in black, respectively. The performances for the stimulus set 1 and set 2 are shown for monkey 1 and monkey 2, respectively. Similar performances were observed in the other stimulus sets (not shown). Blockwise performance for regular stimulus sets is fully reported elsewhere^27^. (**b**) Trialwise behavioral performance before and after the reversal for a regular stimulus set (mean ± SEM). The dataset is the same as that in the black line in (**a**). (**c**) Blockwise behavioral performance before and after the reversal for novel stimulus sets (8 stimuli, mean ± SEM). The left panel shows the performance for the novel stimulus sets in the new learning. The right panel shows the performance before and after the reversal, respectively (monkey 1, n = 17, monkey 2, n = 13). (**d**) Trialwise behavioral performance before and after the reversal for novel stimulus sets. The dataset is the same as that in (**c**).

We first examined whether the macaque monkeys, like Vaughan’s pigeons, were able to efficiently perform the “group reversal” task, in which stimuli predicting either a reward or a punishment were presented trial by trial and occasionally their relationships were reversed (Fig. 1, see Methods), after extensive training. We reversed the stimulus-outcome relationships a number of blocks after they made correct responses to all stimuli in a block. The mean block number to reverse the stimulus-outcome relationships after the behavioral criterion was met was 8.1 ± 0.3 blocks for monkey 1 and 10.1 ± 0.6 blocks for monkey (mean ± SEM, minimum 4 blocks for both monkeys). After an extensive training of the group reversal task using three regular stimulus sets (Fig. 1b), the monkeys learned to quickly adapt to the reversal (black line in the right panel, Fig. 2a). The mean block number that the performance reached the behavioral criterion (correct responses to all the stimuli) after the reversal was 2.0 ± 0.0 blocks in monkey 1 and 2.2 ± 0.1 blocks in monkey 2 (mean ± SEM). Since there was no explicit signal indicating the reversal, the monkeys inevitably made an error at the first trial after the reversal (the correct rate in the first trial after the reversal was 0% in both monkeys. Fig. 2b). However, the monkeys made correct responses to the remaining 7 stimuli in the first block after the reversal in most of the experimental sessions (i.e., 87% in monkey 1 and 91% in monkey 2).

Then, we analyzed how the monkeys’ performance changed in the course of the training sessions. By the time that the monkeys showed the performance in the experimental sessions (black line in Fig. 2a), they had been trained with the regular stimulus sets over 300 sessions from the very first training session. We calculated the average of behavioral performance in every 100 sessions from the first training session (Fig. 2a, orange line: 1st to 100th, green line: 101st to 200th, and cyan line: 201st to 300th sessions). We randomly selected 10 sessions from each division (see Methods). As the training sessions progressed, the performance both in the initial learning and after the reversal gradually improved. The performances in the first (1st to 100th, orange line) and second (101st to 200th, green line) period of the training sessions were, while the performance in the third period of the training sessions (201st to 300th, cyan line) was not, significantly worse than the performance of black line (asterisks in Fig. 2a, t-test, p < 0.05). These results suggest that it took more than 200 sessions, which correspond to several months of training, for the monkeys to learn to quickly adapt their responses to the reversal in the group reversal task.

### Experiment 2

The Vaughan’s procedure was criticized because, after many sessions of the reversal training with a fixed stimulus set, animals might have simply learned to reverse all individual stimulus–response associations at a reversal instead of learning a strategy to use a category for the behavioral adaptation^14,15^. If this is the case, when a novel stimulus set was introduced, it would take many sessions of the reversal training with the stimulus set until the monkeys learn to show a quick adaptation at a reversal. If the monkeys, on the other hand, recognized stimuli associated with the same outcome as a category and utilize the category for the behavioral adaptation at a reversal, they would show a quick adaptation after a reversal even for a novel stimulus set. To respond to the criticisms for the Vaughan’s procedure, we introduced a novel stimulus set (Fig. 1c) in each session and examined how they would perform at the reversal. Figure 2c shows the behavioral performance with novel stimulus sets. The correct rate was about chance level (50%) in the first block, but reached about 90% in the second block (the left panel in Fig. 2c). The mean block number that the performance reached the behavioral criterion (correct responses to all the stimuli) was 2.5 ± 0.2 blocks in monkey 1 and 2.7 ± 0.3 blocks in monkey 2 (mean ± SEM), indicating that the monkeys learned to distinguish between the juice-predicting and saline-predicting pictures after they once or twice experienced the relationship between a picture and its associated outcome for all the pictures. At the reversal, the monkeys showed a quick behavioral adaptation (the right panels in Fig. 2c,d). The mean block number that the performance reached the behavioral criterion after the reversal was 2.4 ± 0.2 blocks in monkey 1 and 2.0 ± 0.0 blocks in monkey 2 (mean ± SEM). These results suggest that the monkeys have learned a strategy to recognize the stimuli predicting the same outcome as a category and used the category to adapt their behavior at the reversal.

### Experiment 3

**Figure 3:**
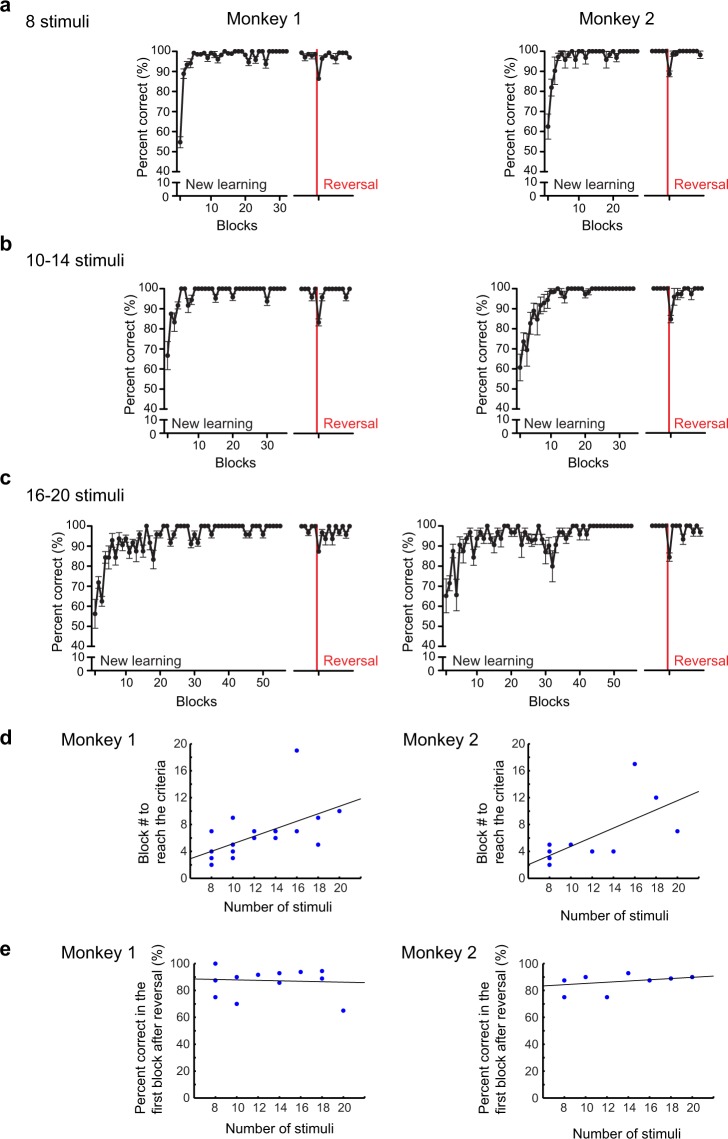
Behavioral performance with stimulus sets with a larger number of stimuli. Blockwise behavioral performances under the conditions with (**a**) 8 novel stimuli (monkey 1, n = 9; monkey 2, n = 8), (**b**) 10–14 novel stimuli (monkey 1, n = 9; monkey 2, n = 3), and (**c**) 16–20 novel stimuli (monkey 1, n = 5; monkey 2, n = 3), respectively (mean ± SEM). (**d**) Number of blocks needed to reach the criterion in the new learnings. The number of blocks that the performance reached the criterion (all correct responses to all stimuli in the stimulus set) was plotted according to the number of stimuli in the stimulus set. The line in each panel is the regression line. Number of data; monkey 1, n = 9, 5, 2, 2, 2, 2, and 1, monkey 2, n = 8, 1, 1, 1, 1, 1, and 1 for 8- stimulus, 10- stimulus, 12- stimulus, 14-stimulus, 16-stimulus, 18-stimulus and 20-stimulus sets, respectively. (**e**) Performance in the first block after the reversal according to the number of stimuli in the stimulus set. The line in each panel is the regression line. The dataset is the same as in (**d**).

We then conducted another experiment to further confirm whether the monkeys reversed their response based on a category, but not on individual stimulus–response associations, at a reversal. We tested the performance of the monkeys with new stimulus sets that contained a larger number of novel stimuli, which was varied from 10 to 20. Since, once a functional category is formed, the memory load to use the category would not be affected by the number of members in the category^16^, we predicted that the monkeys’ performance at the reversal would not change even when the number of stimuli to be remembered increased if the monkeys had formed a category for the stimuli associated with the same outcome. In contrast, if the monkeys based their performance on the individual stimulus–outcome associations, their performance at the reversal would deteriorate as the number of stimuli to be remembered increased. Figure 3 shows the behavioral performance under the conditions with the stimulus sets with a large number of stimuli. For the new learning, the monkeys needed a larger number of trial blocks to reach the behavioral criterion (responding correctly to all the stimuli in a block), as the number of stimuli in a set increased. We found significant positive correlations between the number of stimuli and the number of blocks that the monkeys needed to reach the criterion (Fig. 3d, monkey 1, r = 0.59, df = 21, *p* = 0.003; monkey 2, r = 0.69, df = 12, *p* = 0.006, Pearson’s correlation coefficient). After the reversal, however, the monkeys quickly adapted their behavior regardless of the number of stimuli. There was no significant correlation between the performance in the first block after the reversal and the number of stimuli in the stimulus set (Fig. 3e, monkey 1, r = −0.08, df = 21, *p* = 0.70; monkey 2, r = 0.33, df = 12, *p* = 0.26, Pearson’s correlation coefficient). These results suggest that the monkeys, rather than learning the individual stimulus–outcome association, learned and used a category for the stimuli associated with the same outcome.

### Experiment 4

**Figure 4:**
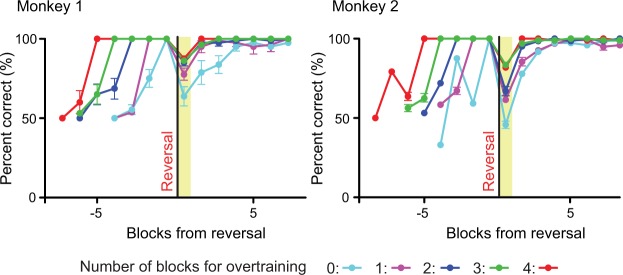
Behavioral performances before and after the reversals at different times of onset (mean ± SEM). Reversals occurred at different times of onset after the behavioral performance reached the criterion. The criterion was that the monkey made correct responses to all of the 8 novel stimuli in a block. “0-block” means the reversal occurred immediately after the criterion was reached. “1-block”, “2-block”, “3-block”, and “4-block” mean the reversal occurred after 1, 2, 3, and 4 additional blocks under the original condition after the criterion was reached, respectively. Number of sessions that were used for the plot is as follows; monkey 1, n = 10, 10, 10, 10, and 5, monkey 2, n = 8, 12, 8, 12, and 11 for 0-block, 1-block, 2-block, 3-block, and 4-block overtraining sessions, respectively.

So far, we have shown that the monkeys learn to use a category in a group reversal task. In Experiment 4, to examine when the category formation is completed after the introduction of a new stimulus set, we introduced a novel stimulus set in each session and reversed the condition at different times of onset after the behavioral performance reached the criterion (correct responses to all of 8 stimuli in a block). In training sessions, we usually reversed the condition in the fifth (“4-block” overtraining) or later block after the behavioral criterion was met. Figure 4 shows the behavioral performance after the reversal at different times of onset. We compared the correct rate in the first block after the reversal according to the reversal timings (“0-block”, “1-block”, “2-block”, “3-block”, and “4-block” overtraining, which we reversed the condition in the first, second, third, fourth, and fifth block after the criterion was met, respectively) by a repeated measure ANOVA with a Tukey’s post-hoc test. We found a significant main effect in both monkeys (monkey 1, *F*_(4, 40)_ = 6.57, *p* = 0.0004; monkey 2, *F*_(4, 46)_ = 8.94, *p* < 10^−4^). The post-hoc test revealed that the correct rate in the first block after the reversal was significantly different between “0-block” overtraining session and “2-block” overtraining session in monkey 1 (*p* = 0.0019). The post-hoc test also revealed that the correct rate in the first block after the reversal was significantly different between “0-block” overtraining session and “3-block” overtraining session, and between “0-block” overtraining session and “4-block” overtraining session in both monkeys (monkey 1, *p* = 0.0002 for “0-block” vs. “3-block”, *p* = 0.0001 for “0-block” vs. “4-block”; monkey 2, *p* = 0.001 for “0-block” vs. “3-block”, *p* = 0.0054 for “0-block” vs. “4-block”). The mean number of blocks that the behavioral performance reached the criterion after the reversal was as follows; monkey 1, 3.8 ± 0.6, 2.3 ± 0.2, 2.4 ± 0.3, 2.3 ± 0.2, and 2.0 ± 0.0 blocks for “0-block”, “1-block”, “2-block”, “3-block”, and “4-block” overtraining, respectively (mean ± SEM); monkey 2, 4.3 ± 0.9, 3.4 ± 0.5, 2.4 ± 0.3, 2.3 ± 0.2, and 2.0 ± 0.0 blocks. These results indicate that a category seems to be formed after a few blocks, once the monkeys correctly responded to all of the stimuli.

## Discussion

In Experiments 1, 2 and 3, we examined whether macaque monkeys (Japanese monkeys) were able to form and utilize functional category (equivalence classes) by using a repetitive group reversal, which is a procedure originally used by Vaughan for pigeons^9^. The results indicate that monkeys can form and utilize functional category and support Vaughan’s claim that equivalence classes can be learned through repetitive reversal learning.

In Experiments 1 and 2, we found that the monkeys showed a rapid behavioral change at the reversal, after they learned the stimulus–outcome associations, in the group reversal task. They showed a rapid adaptation to not only regular stimulus sets, with which they were overtrained for over several months, but also novel stimulus sets, which were introduced in daily sessions (Fig. 2c). They usually reversed their responses after a single error at the reversal and made correct responses to the remaining stimuli even for the first time that they experienced the change in the association for those stimuli. This result suggests that a new contingency in one member of the stimulus group was transferred to the other members of the group, which in turn suggests that the monkeys used category to perform the group reversal task.

In Experiment 3, we examined the effects of increasing memory load on the performance of the group reversal task by increasing the number of cue stimuli. If the monkeys learned to perform the task on the basis of individual stimulus–outcome associations, the performance would decrease as the memory load increased. In contrast, if the monkeys learned and used a category to perform the task, the performance would not change even if the number of cue stimuli increased because categorizing stimuli into subgroups can relieve the memory load^16^. The monkeys could quickly adapt their behavior to the new stimulus–outcome contingencies at the reversal even when the number of stimuli to be remembered increased, although the monkeys needed more trials in the new learning as the number of stimuli increased (Fig. 3). This result also supports the view that the monkeys, rather than learning the individual stimulus–outcome associations, learned to form and utilize category to perform the group reversal task.

In Experiment 4, we studied when the category formation is completed after the introduction of a new stimulus set by changing the time of onset of reversals. Monkeys showed a rapid adaptation to a reversal with a few blocks of overtraining after the performance reached the behavioral criterion (i.e., responding correctly to all the stimuli in a block). However, they showed a slower adaptation to a reversal introduced immediately after the criterion was met. The results suggest that monkeys first learn the individual stimulus–outcome associations and the formation of the category is completed in a few blocks after they correctly responded to all of the stimuli.

Previous studies have shown that not only pigeons^10^ but also other species, such as dolphins^11^, sea lions^12^, and chimpanzees^13^ can be trained to efficiently perform the group reversal task with familiar stimuli. This study has shown that monkeys can learn to perform the group reversal task efficiently with familiar stimuli, and furthermore, they are capable of applying the strategy acquired through extensive training to a new situation with novel stimuli, which indicates that they are capable of forming and using functional category in general.

How is a category formed? As the results of Experiment 4 showed, the monkeys first learned individual stimulus-response associations, then formed and used a category after a few blocks. While the monkeys experienced individual stimulus-outcome relations a few times, the stimuli that were associated with the same outcome may be grouped together, forming stimulus-stimulus associations. Such stimulus-stimulus associations may be the basis of a category.

There have been criticisms for Vaughan’s procedure, claiming that animals may learn individual stimulus-response associations and reverse their response based on the individual associations at a reversal^14,15^. Although the monkeys showed a quick adaptation after a reversal in the sessions with a novel stimulus set (Experiment 2) and in the sessions with a larger number of stimuli (Experiment 3), it is still possible in a theoretical sense that they learned individual stimulus-outcome associations and reversed their response based on the individual associations at a reversal. However, as the number of stimuli increases, it becomes increasingly difficult to respond individually rather than to respond based on a category. It is very likely that the monkeys formed and used a category to perform the task efficiently. Previous studies have reported that neurons in the macaque prefrontal cortex, lateral cortex, and striatum show similar responses to stimuli that have the same meaning to the subject (i.e., functional equivalent stimuli), suggesting that there are neural correlates of a category in the monkey’s brain^17–25^.

The evidence that monkeys can learn to use category through a simple procedure (i.e., repetitive group reversals) is important. Macaque monkeys are widely used in neurophysiological studies; thus, we can study neural mechanisms underlying the formation and usage of category in monkeys by using a group reversal task. We have recently recorded neuronal activity in the prefrontal cortex while the monkeys were performing the same task used in this study, and found that a subset of prefrontal neurons code category information^26^. It is expected that, by using the group reversal task, the neural mechanisms of category formation and utilization would be studied extensively, which may lead us to deeper understandings of higher cognitive functions.

## Methods

### Subjects

Two naïve male Japanese monkeys (3 and 9 years of age at the time of the experiments, *Macaca fuscata*) were used as experimental subjects. Throughout the experiments, they were treated in accordance with the National Institutes of Health Guide for the Care and Use of Laboratory Animals and the Tohoku University Guidelines for Animal Care and Use. This project was approved by the Center for Laboratory Animal Research of Tohoku University. Monkeys were housed individually in a cage in a room with natural lighting, and were fed a normal diet (such as commercial monkey-chow and fruits) twice a day with *ad libitum* access to water. The experiments were conducted before the monkeys were fed a diet.

### Apparatus

In the laboratory, a monkey sat on a home-made primate chair to which a touch-key sensor (Supertech, Hungary) was attached. Visual stimuli were presented at the eye level on a liquid-crystal display (LCD) (Prolite E431S, Iiyama, Japan) placed 35 cm in front of the monkey. A double-spout device, through which two types of liquid were delivered as reward and punishment, was placed in front of the monkey’s mouth. An infrared sensor (Supertech, Hungary) was attached to the spout device to monitor the monkey’s spout-licking behavior. We used abstract figures for visual stimuli because they had no intrinsic meaning for the monkeys. All of the abstract figures used as visual stimuli had been generated as computer graphics files (bitmap format, 142 × 142 pixels) and stored on a hard disk. The size of a visual stimulus extended to 6° × 6° in visual angle when it was presented on the LCD. Orange juice (Torys Conc, Suntory) was used as a reward and concentrated saline (7%) was used as a punishment. Visual stimulus presentation was controlled using graphics presentation software (Presentation, Neurobehavioral Systems) in a personal computer (xSeries100, IBM Japan, Japan) that was synchronized with and controlled by a home-made host computer. Juice/saline delivery was controlled by the opening of solenoid valves (CKD Corporation, Japan) remotely controlled by the host computer.

### Behavioral task

Monkeys performed the “group reversal” task in which they had to adapt their behavior to a repeated stimulus–outcome reversal with a set of eight distinct visual stimuli and two distinct outcomes (Fig. 1). In every trial, a visual stimulus was presented to the subject, and then its associated outcome (juice as reward or saline as punishment) was delivered through the spout in front of the mouth. Therefore, the stimulus served as a cue to predict the outcome of the trial. The correct response at the time of liquid delivery was to lick when the preceding stimulus had been associated with juice (reward acquisition by the “go” response) and not to lick when it had been associated with saline (punishment avoidance by the “no-go” response). If the monkey did not lick the liquid, it dropped to the tray that was equipped under the spout. The tray was not accessible by the monkey. The precise time sequence of the task events was as follows. When a red fixation spot (4 mm in diameter) appeared at the center of the LCD, the monkey touched the key and fixated on the spot. After a precue period (either varied between 1.0 and 1.5 s or fixed at 1.25 s), a visual stimulus was presented at the center of the LCD for 0.75 s. After the cue offset, a delay period lasted for either a duration varied between 0.75 and 1.5 s or fixed at 1.25 s. When the fixation spot turned from red to green, the monkey released the key, and the fixation spot disappeared. After the key release, another delay period lasted for 0.5 s then either juice or saline was delivered. For the precue and first delay periods, the timing of stimulus presentation was varied during the training sessions. Later, the timing was fixed. We counted a trial as correct if a monkey licked the spout within a certain time window including the outcome delivery time (from 200 ms before the delivery onset to the end of the delivery) in juice trials and did not lick the spout within the time window in saline trials. We repeated the same trial (correction trial) if a monkey terminated a trial erroneously by breaking its fixation or releasing the key early until the monkey learned to complete a trial even when the predicted outcome was saline. In each trial, a stimulus was pseudorandomly selected from the stimulus set in such a way that each stimulus in a set was presented once in a block of eight trials. Four of the stimuli in a set were associated with juice and the other four were associated with saline, and occasionally the association relationships were all reversed without an explicit cue (rule reversal). We labeled one of the four-stimulus groups “group A” and the other “group B”. No explicit external cue was given to indicate the occurrence of the rule reversal, but the first trial after the reversal was always a saline trial so that a monkey would notice the reversal. Therefore, the punishment (an unexpected delivery of saline in the first trial after the rule reversal) served as a cue for the rule reversal. Even when the stimulus–outcome relationship was reversed in the rule reversal, the functional equivalence of each of the four stimuli in a group was always preserved (i.e., they predicted the same outcome) and thus constituted a functional category. Eye movements were monitored using an infrared eye movement recording system (ETL-200, I-scan, USA). The trial was canceled immediately when the eye position exceeded the limit of 1° from the fixation spot. The eye movements during each trial were also examined offline to confirm eye fixation.

In Experiment 3, we used stimulus sets with more than eight stimuli. We incremented the number of stimuli by two from 10 to 20, while keeping the number of stimuli the same between groups A and B. In daily sessions, we started an experiment with one of the regular stimulus sets (eight stimuli) and confirmed that the monkey was motivated to perform the task. Then, we introduced a novel stimulus set with more than eight stimuli. We randomly selected the number of stimuli in the stimulus set.

### Statistics

In experiment 1, to study how the monkey’s performance changed in the course of the training sessions, we divided the data of the 300 training sessions into 3 divisions (i.e., 1st~100th, 101st~200th, and 201st~300th sessions), and compared the average performance, separately for each block, between the experimental sessions and each division of the training sessions, using one-tailed t-test (the significance level was set at 0.05). We randomly selected 10 sessions from each division, and compared the performance.

In experiment 4, to study when the category formation was completed after the introduction of a new stimulus set, we reversed the condition at different times of onset after the behavioral performance reached the criterion (correct responses to all of 8 stimuli), and compared the average performance in the first block after the reversal between the sessions with different onsets of reversal by a repeated measure ANOVA with a Tukey’s post-hoc test (the significance level was set at 0.05).
